# Prognostic thresholds of outcome predictors in severe accidental hypothermia

**DOI:** 10.1007/s11739-024-03741-1

**Published:** 2024-09-12

**Authors:** Konrad Mendrala, Tomasz Darocha, Tomáš Brožek, Sylweriusz Kosiński, Martin Balik, Evelien Cools, Beat Walpoth, Ewelina Nowak, Wojciech Dąbrowski, Bartosz Miazgowski, Kacper Reszka, Aleksander Rutkiewicz, Guillaume Debaty, Nicolas Segond, Michał Dudek, Stanisław Górski, Paweł Podsiadło

**Affiliations:** 1 Department of Anaesthesiology and Intensive Care, Medical University of Silesia, Katowice, Poland; 2 Department of Anaesthesiology and Intensive Care, 1st Faculty of Medicine, Charles University and General University Hospital, Prague, Czech Republic; 3 Department of Intensive Interdisciplinary Therapy, Jagiellonian University Collegium Medicum, Krakow, Poland; 4 Department of Acute Medicine, Division of Anaesthesiology, University Hospitals, Geneva, Switzerland; 5 Emeritus. Department of Cardiovascular Surgery, University Hospitals of Geneva, Geneva, Switzerland; 6 Institute of Health Sciences, Jan Kochanowski University, Kielce, Poland; 7 Department of Anaesthesiology and Intensive Care, Medical University of Lublin, Lublin, Poland; 8 Emergency Department, University Hospital, Pomeranian Medical University, Szczecin, Poland; 9 Department of Anaesthesiology and Intensive Care, University Hospital, Łódź, Poland; 10 Department of Anaesthesiology and Intensive Care, Cieszyn, Poland; 11 Univ. Grenoble Alpes, CNRS, UMR 5525, VetAgro Sup, Grenoble INP, CHU Grenoble Alpes, TIMC, 38000 Grenoble, France; 12 Department of Emergency Medicine, Faculty of Health Sciences, University of Bielsko, Biała, Poland; 13 Department of Medical Education, Jagiellonian University Medical College, Kraków, Poland; 14 Department of Emergency Medicine, Jan Kochanowski University, Kielce, Poland

**Keywords:** Emergency medicine, Hypothermia, Hypotension, Rewarming, Death, Risk factors

## Abstract

Hemodynamically unstable patients with severe hypothermia and preserved circulation should be transported to dedicated extracorporeal life support (ECLS) centers, but not all are eligible for extracorporeal therapy. In this group of patients, the outcome of rewarming may sometimes be unfavorable. It is, therefore, crucial to identify potential risk factors for death. Furthermore, it is unclear what criterion for hemodynamic stability should be adopted for patients with severe hypothermia. The aim of this study is to identify pre-rewarming predictors of death and their threshold values in hypothermic patients with core temperature ≤ 28 °C and preserved circulation, who were treated without extracorporeal rewarming. We conducted a multicenter retrospective study involving patients in accidental hypothermia with core temperature 28 °C or lower, and preserved spontaneous circulation on rewarming initiation. The data were collected from the International Hypothermia Registry, HELP Registry, and additional hospital data. The primary outcome was survival to hospital discharge. We conducted a multivariable logistic regression and receiver operating characteristic curve (ROC) analysis. In the multivariate analysis of laboratory tests and vital signs, systolic blood pressure (SBP) adjusted for cooling circumstances and base excess (BE) were identified as the best predictor of death (OR 0.974 95% CI 0.952–0.996), AUC ROC 0.79 (0.70–0.88). The clinically relevant cutoff for SBP was identified at 90 mmHg with a sensitivity of 0.74 (0.54–0.89) and a specificity of 0.70 (0.60–0.79). The increased risk of death among hypothermic patients with preserved circulation occurs among those with an SBP below 90 mmHg and in those who developed hypothermia in their homes.

## Introduction

Accidental hypothermia (AH) is a heterogeneous entity, and the decision of rewarming method depends mainly on the clinical situation. According to the European Resuscitation Council (ERC) 2021 guidelines, patients with cardiac arrest should be transported to a specialized center with ECLS (extracorporeal life support) therapy, and the chance of favorable outcome should be assessed using the Hypothermia Outcome Prediction after Extracorporeal life support score (HOPE) [1, 2]. Hemodynamically unstable patients with preserved circulation should be transported to dedicated ECLS centers [3]. However, the final rewarming method is dictated by an individual assessment of the patient, coexisting injuries, comorbidities, frailty, and functional dependence prior to hypothermia.

Rewarming patients with preserved circulation using extracorporeal membrane oxygenation (ECMO) is a highly invasive procedure and should be limited to whom the benefit outweighs the risk of complications, and implementation of this therapy may give the potential for hospital discharge. Therefore, despite the relative indications, not all patients qualify for this type of rewarming. In such cases, non-invasive methods (forced air warming, warming mattress, infrared heating) and invasive methods (peritoneal-, pleural-, bladder-, and stomach- warm lavage, intravascular heating systems, kidney support therapy) remain an option [4].

Some patients undergoing non-ECLS rewarming may be deemed ineligible for ECMO but die during rewarming process. Therefore, it is crucial to identify potential risk factors of death in this group of patients. Moreover, it is not clear what criterion of hemodynamic stability should be adopted for patients in hypothermia with core temperature ≤ 28 °C and preserved circulation in whom significantly reduced tissue metabolism offers the possibility of a more liberal approach for maintaining organ perfusion. The ERC indicates systolic blood pressure (SBP) < 90 mmHg and/or ventricular arrhythmias as criteria for unstable patients. The Wilderness Medical Society Clinical Practice Guidelines discuss the concern of hemodynamic instability, but does not define it. Our previous study identified blood pressure, PaCO2, circumstances of hypothermia and co-morbidities as factors affecting the outcome of rewarming. However, specific values for these variables characterizing patients at increased risk of death were not identified.

## Aims

The aim of this study is to identify pre-rewarming predictors of death and their threshold values in hypothermic patients with core temperature ≤ 28 °C and preserved circulation, who were treated without extracorporeal rewarming.

## Material and methods

The study received Research Ethics Approval from Medical University of Silesia (no PCN/CBN/0052/KB/32/23). It is designed as a retrospective, observational, and multicenter study of AH patients. We used individual patient data collected for the study by Podsiadło et al. [5]. The data was collected from the International Hypothermia Registry (IHR), Hypothermia Life Support in Poland (HELP) Registry (https://rejestrhipotermii.ujk.edu.pl/), and the hospitals involved in that study. The data have been updated up to 1st April 2023.

The primary outcome was survival to hospital discharge.

### Inclusion criteria

Adult patients > 18yo < 90yo with accidental hypothermia, core temperature of ≤ 28 °C, and preserved spontaneous circulation at patient discovery and at rewarming commencement were included in the analysis. All patients underwent non-ECLS rewarming.

### Exclusion criteria

Exclusion criteria were: hypothermia associated with asphyxia (drowning, avalanche victims); cardiac arrest without return of spontaneous circulation; severe trauma with hemorrhagic shock and other non-hypothermia-related hemodynamic instability; implanted pacemaker. Also, patients with terminal illnesses and receiving palliative treatment were excluded from analysis.

### Data collection

The following data were collected: Patient age, gender, comorbidities, circumstances of hypothermia development (indoors/outdoors), vital signs at hospital admission (core temperature, heart rate, blood pressure, ventricular arrhythmias), occurrence of cardiac arrest with return of spontaneous circulation (ROSC) at any time of patient’s management before rewarming, mechanical ventilation before rewarming, and laboratory tests on admission (arterial blood gases with no temperature correction, acid–base balance, potassium and lactate concentration). When the non-invasive blood pressure was reported as „unmeasurable”, we assigned the value of 30 mmHg to such cases because this is the lowest systolic blood pressure measured by cardiac monitors commonly used in emergency departments.

### Data processing and analysis

Initially, we compared the surviving patients with the deceased group to identify variables associated with unfavorable outcome. The distribution of the variables was assessed using the Shapiro–Wilk test and QQ plots. Differences between groups were assessed with the Pearson’s chi-squared test, Mann–Whitney U test, or Student's t- test, depending on the variable’s distribution. In the descriptive statistics, variables are presented as mean and 95%CI or median and IQR. Qualitative variables are presented as absolute values and percentages.

We also performed a post-hoc analysis assessing the relationship between comorbidities (Charlson Comorbidity Index, CCI) and cooling circumstances to check whether the latter parameter could substitute for CCI [6]. Further, we developed a multivariable logistic regression. The potential risk factors were chosen based on previously published research in hypothermic patients: cooling circumstances, age, gender, core temperature (Tc), heart rate (HR), systolic blood pressure (SBP), diastolic blood pressure (DBP), mean arterial pressure (MAP), alpha-stat arterial blood gases at admission (temperature uncorrected), lactate and potassium concentrations, CCI, catecholamines administration, mechanical ventilation. We converted the PaCO_2_ originally measured with the alpha-stat method to pH–stat using the following formula [7]:

$$pH \, PaCO2 = alpha \times PaCO2 \times 10EXP(0.021(temperature - 37)) \,$$

Spearman correlation coefficients were determined, and only variables with correlations < 0.7 were included in the analysis. Univariate logistic regression was performed, based on which the independent variables with the highest OR/value of the Wald test were selected at the level of significance 0.25. We conduct a purposeful selection of variables as per Bursac et al. [8]. In the binominal regression model, significance of variables was determined at the 0.1 alpha level, while confounding was defined as a change in the remaining parameter of more than 20%. When covariates were non-significant and not cofounders, they were eliminated from the model. Model evaluation was based on the Hosmer–Lemeshow test, and Negelkerke R Square. The comparison of the models was based on the AUC and the coordinates of the ROC curve.

Finally, we calculated the cut-off values of risk factors with their sensitivity and specificity values. For statistical analysis we used StatsDirect 3.3.5 (StatsDirect LTD, Wirral, UK).

## Results

**Fig. 1:**
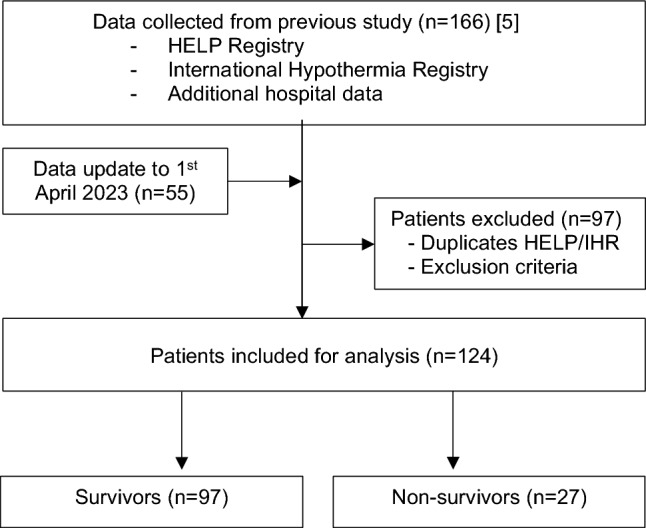
Flowchart of data collection for analysis

A total of 124 patients (99 males and 25 females, aged between 18 and 89 years) were analyzed. The data collection flowchart is shown in Fig. 1. The compared groups did not differ in Tc. The lowest Tc was 20.8 °C which was also the lowest Tc in a survivor. Thirty-four patients (27.4%; 34/124) were found indoors, seventy-seven (62.1%; 77/124) outdoors, in thirteen patients (10.5%; 13/124) the location of hypothermia occurrence was not determined. Calculated Charlson Comorbidity Index was higher in non-survivors (3 vs 2pts). Similar, indoor cooling was significantly associated with higher CCI (3 vs 2pts).

Of the 124 patients, 27 died (21,8%); this group was characterized by lower arterial blood pressure (for SBP 69 vs 100 mmHg), lower paCO2 values on arterial blood gas analysis (for alpha stat 40 vs 49 mmHg), and older age (64 vs 56 years). Six patients had unmeasurable SBP (substituted by 30 mmHg), half of whom survived. A significant risk factor for death was the localization of cooling. Patients found indoors were more likely to die than patients found outdoors (52.9% vs 10.4%). Detailed data are available in Table 1.

**Table 1:** Univariate analysis of risk factors for death in HT3 patients not qualified for extracorporeal rewarming

	n	Survivors	Non-survivors	n	p	Univariate binominal logistic regression		
						OR (death)	p	AUC ROC
SBP (mmHg)	97	100.4 (94.1–106.7)	68.7 (59.1–78.4)	27	< 0.001*	0.963 (0.946–0.980)	** < 0.001**	0.79
DBP (mmHg)	95	57 (53–61)	44.9 (38.7–51.1)	24	0.005*	0.966 (0.942–0.991)	**0.007**	0.70
MAP (mmHg)	94	72.2 (68.1–76.3)	55.6 (48.8–62.5)	23	< 0.001*	0.955 (0.929–0.981)	** < 0.001**	0.75
HR (mmHg)	95	50 (40–65)	46.5 (40–60)	26	0.399	0.993 (0.970–1.016)	0.547	0.55
Tc (°C)	97	26.3 (25.3–27.0)	26.6 (25.1–27.2)	27	0.607	1.112 (0.842–1.469)	0.441	0.53
pH	79	7.22 (7.142–7.295)	7.21 (7.06–7.29)	21	0.898	0.281 (0.013–5.992)	0.416	0.51
paO_2_ (mmHg)	78	126 (78.1–245.4)	112 (78–155)	21	0.294	0.997 (0.992–1.002)	0.201	0.57
paCO_2_ (mmHg)								
Alpha-stat	79	49.0 (41.2–54)	40.3 (30.9–45.4)	21	**0.013**	0.963 (0.927–1.001)	0.058	0.68
pH–stat	79	29.1 (23.3–32.25)	21.5 (18.47–28.96)	21	**0.002**	0.947 (0.891–1.007)	0.084	0.67
BE (mmol/L)	74	-7.45 (-11.2- -3.1)	-6.9 (-16.6- -4.1)	21	0.297	0.949 (0.893–1.008)	0.088	0.58
HCO_3_ (mmol/L)	65	20.8 (17.1–24)	21.3 (15.7–22.4)	17	0.608	0.961 (0.887–1.041)	0.326	0.54
K^+^ (mmol/L)	86	3.65 (3.1–4.1)	3.8 (3.3–4.14)	23	0.611	1.001 (0.597–1.679)	0.997	0.53
Lac (mmol/L)	85	4.3 (1.8–7.3)	3.4 (1.7–5.9)	22	0.607	0.988 (0.885–1.103	0.831	0.54
AGE (years)	97	56.4 (53.6–59.2)	63.5 (58–69.1)	27	0.021*	1.039 (1.005–1.073)	**0.024**	0.65
INDOOR	85	16 (18.8%)	18 (69.2%)	26	** < 0.001**	9.703 (3.589–26.236)	** < 0.001**	–
CCI	83	2 (1–3)	3 (2–4)	22	** < 0.001**	0.687 (0.540–0.874)	0.002	0.73
MALE	97	77 (79.4%)	22 (81.5%)	27	0.810	1.143 (0.385–3.394)	0.810	–
MV	95	27 (28.4%)	16 (38.5%)	26	0.325	1.574 (0.635–3.9)	0.327	–
CA ROSC	93	9 (9.7%)	4 (16%)	25	0.47	1.778 (0.499–6.338)	0.375	–
VAs	17	2 (2.1%)	0 (0%)	1	–	–	–	–

Bold values indicate statistically significant results

Data are presented as mean and 95%CI or as median and IQR. Qualitative data are presented as absolute value and %

*SBP* systolic blood pressure, *DBP* diastolic blood pressure, *MAP* mean arterial pressure, *HR* heart rate, *Tc* core temperature, *BE* base excess, *Lac* lactates, *CCI* Charlson Comorbidity Index, *MV* mechanical ventilation, *CA ROSC* cardiac arrest with return of spontaneous circulation, *VAs* ventricular arrhythmias, *n* reported data

*t-Student

**Fig. 2:**
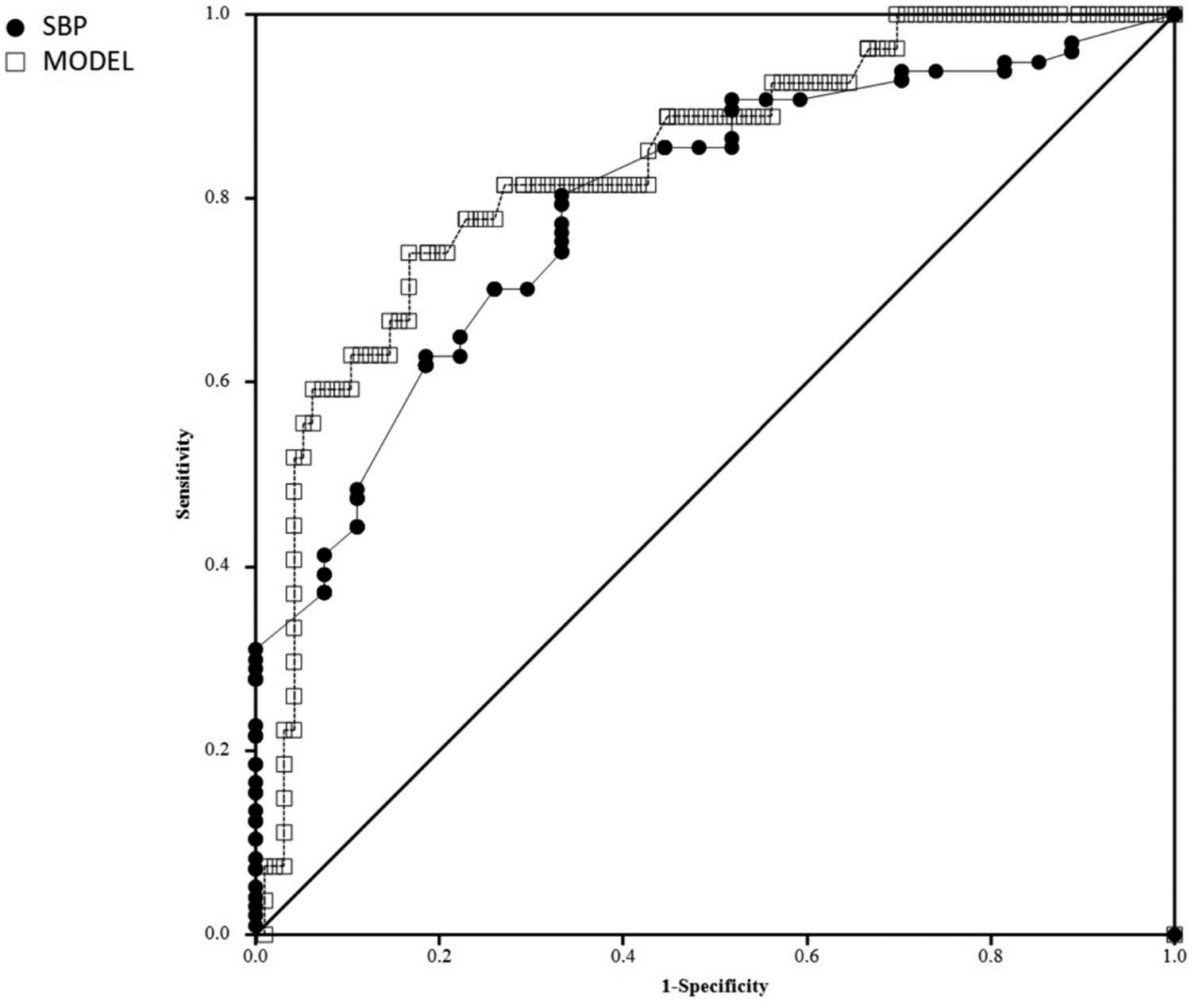
Performance of a classification model. The curves with the largest area under the curve are shown. Model (SBP/exposure/BE) Wilcoxon estimate of area under ROC curve 0.84 (0.75–0.92); SBP Wilcoxon estimate of area under ROC curve 0.79 (0.70–0.88)

**Fig. 3:**
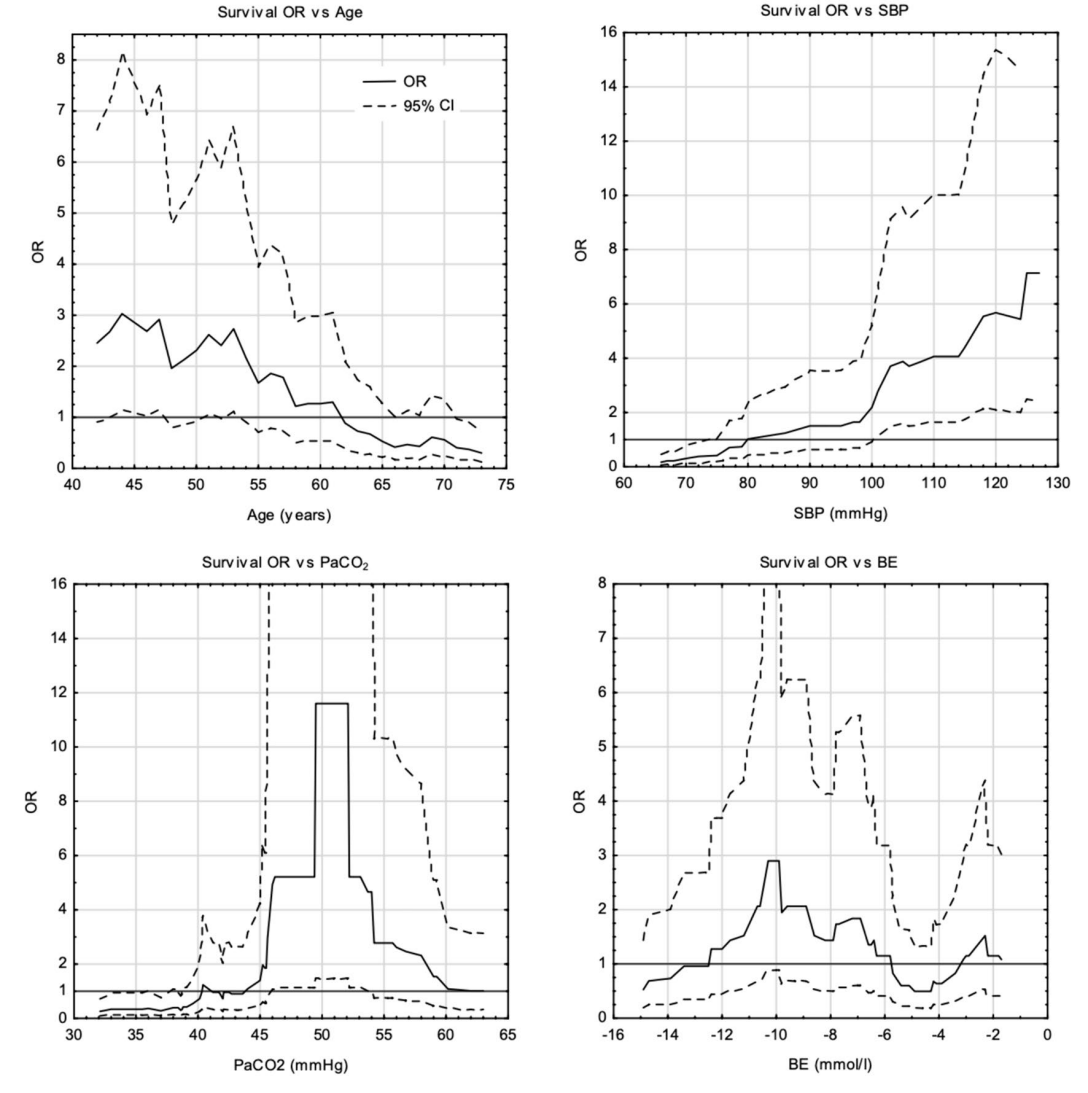
Graphs show the relationship between OR of survival and the values of tested variables. For paCO_2_ and BE a direct effect on mortality was not found

In the multivariate analysis of laboratory tests and vital signs, SBP adjusted for cooling circumstances and BE was identified as the best predictor of death (OR 0.974 95% CI 0.952–0.996), AUC ROC 0.79 (0.70–0.88), Table 2. The clinically relevant cutoff for SBP was identified at 90 mmHg with sensitivity 0.74 (0.54–0.89) and specificity 0.70 (0.60–0.79), Figs. 2, 3. Cut-off values for selected arterial pressures and age are shown in Table 3. For paCO2 and BE a direct effect on mortality was not found. Graphs showing OR against the values of the variables are shown in Fig. 3.

**Table 2:** Potential death risk factors model based on multivariate binary logistic regression

	Wald	B	OR	p
SBP (mmHg)	5.208	− 0.027	0.974 (0.952–0.996)	0.022
Indoor cooling circumstances	12.166	2.365	10.644 (2.818–40.202)	< 0.001
BE (mmol/L)	2.849	− 0.068	0.934 (0.863–1.011)	0.091
Constant	0.344	− 0.665	0.514	0.557

Hosmer and Lemeshow Test 0.18; Nagelkerke R Square 0.47; -2 Log likelihood 62.94; Cox & Snell R Square 0.316

**Table 3:** Cutoffs for selected blood pressure values and age. Values are given with 95%CI

Cutoff	Sensitivity	Specificity	post-test likelihood of death (%)	post-test likelihood of survival (%)	post-test death likelihood despite negative test (%)
SBP (mmHg)					
60	0.41 (0.22–0.6)	0.91 (0.82–0.96)	55 (31.5–76.9)	84.6 (76.2–90.9)	15.4 (9.1–23.8)
65	0.48 (0.29–0.68)	0.86 (0.77–0.92)	48.2 (28.7–68.1)	85.6 (77.0–91.9)	14.4 (8.1–23.0)
70	0.67 (0.46–0.83)	0.80 (0.71–0.88)	48.7 (31.9–65.6)	89.7 (81.3–95.2)	10.3 (4.8–18.7)
75	0.67 (0.46–0.83)	0.77 (0.67–0.85)	45 (29.3–61.5)	89.3 (80.6–95.0)	10.7 (5.0–19.4)
80	0.70 (0.50–0.86)	0.70 (0.60–0.79)	39.6 (25.8–54.7)	89.5 (80.3–95.3)	10.5 (4.7–19.7)
85	0.70 (0.46–0.83)	0.70 (0.71–0.88)	48.7 (31.9–65.6)	89.7 (81.3–95.1)	10.3 (4.8–18.7)
90	0.74 (0.54–0.89)	0.70 (0.60–0.79)	40.8 (27–55.8)	90.7 (81.7–96.1)	9.3 (3.8–18.3)
95	0.77 (0.58–0.91)	0.63 (0.52–0.72)	36.8 (24.5–50.7)	91 (81.5–96.6)	8.9 (3.4–18.5)
100	0.89 (0.71–0.98)	0.48 (0.38–0.59)	32.4 (22–44.3)	94 (83.5–98.8)	6 (1.3–16.6)
MAP (mmHg)					
45	0.26 (0.10–0.48)	0.93 (0.85–0.97)	46.2 (19.2–74.9)	83.7 (75.1–90.2)	16.4 (9.8–24.9)
50	0.52 (0.31–0.73)	0.83 (0.74–0.90)	42.7 (24.5–62.8)	87.6 (79.0–93.7)	12.4 (6.3–21.0)
55	0.61 (0.39–0.80)	0.78 (0.68–0.86)	40 (23.9–58.0)	89.0 (80.2–94.9)	11.0 (5.1–19.8)
60	0.61 (0.39–0.80)	0.69 (0.59–0.78)	32.6 (19.1–48.5)	87.8 (78.2–94.3)	12.2 (5.7–21.8)
65	0.70 (0.47–0.87)	0.64 (0.53–0.73)	32 (19.5–46.7)	89.6 (79.7–95.7)	10.5 (4.3–20.4)
70	0.74 (0.52–0.90)	0.56 (0.46–0.67)	29.3 (18.1–42.7)	89.8 (79.2–96.2)	10.2 (3.8–20.8)
75	0.87 (0.66–0.97)	0.46 (0.35–0.56)	28.2 (18.1–40.1)	93.5 (82.1–98.6)	6.52 (1.4–17.9)
80	0.91 (0.72–0.99)	0.36 (0.27–0.47)	25.9 (16.8–36.9)	94.4 (81.3–99.32)	5.6 (0.7–18.7)
DBP (mmHg)					
35	0.25 (0.09–0.47)	0.87 (0.79–0.93)	33.3 (13.3–59.0)	82.2 (73.3–89.0)	17.8 (10.9–26.7)
40	0.5 (0.29–0.71)	0.77 (0.67–0.85)	35.3 (19.8–53.5)	85.9 (76.6–92.5)	14.1 (7.5–23.4)
45	0.63 (0.41–0.81)	0.69 (0.59–0.79)	34.1 (20.5–49.9)	88 (78.4–94.4)	12 (5.6–21.6)
50	0.67 (0.45–0.84)	0.6 (0.49–0.70)	29.6 (18.0–43.6)	87.7 (77.2–94.5)	12.3 (5.5–22.8)
55	0.71 (0.49–0.87)	0.59 (0.48–0.69)	30.4 (18.8–44.1)	88.9 (78.4–95.4)	11.1 (4.6–21.6),
60	0.88 (0.68–0.97)	0.4 (0.30–0.51)	26.9 (17.5–38.2)	92.7 (80.1–98.5)	7.32 (1.5–19.9)
AGE (years)					
55	0.78 (0.58–0.91)	0.44 (0.34–0.55)	28 (18.2–39.6)	87.8 (75.2–95.4)	12.2 (4.6–24.8)
60	0.63 (0.42–0.81)	0.57 (0.46–0.67)	28.8 (17.8–42.1)	84.6 (73.5–92.4)	15.4 (7.6–26.5)
65	0.59 (0.39–0.78)	0.71 (0.61–0.80)	36.4 (22.4–52.2)	86.3 (76.7–92.9)	13.8 (7.1–23.3)
70	0.33 (0.17–0.54)	0.84 (0.75–0.90)	36 (18.0–57.5),	81.8 (72.8–88.9)	18.2 (11.2–27.2)

## Discussion

In clinical practice clear recommendations regarding qualification for ECLS in severely hypothermic patients who have detectable vital signs and do not require chest compressions are lacking, therefore we investigated this topic more thoroughly. Our study shows that among severely hypothermic, non-CA patients at high risk of death are those with systolic blood pressure < 90 mmHg and those who developed hypothermia in their homes. This result is of particular importance rationalizing the use of the 90 mmHg SBP threshold as one of the hemodynamic instability criteria in the ERC guidelines [3]—value commonly found in the literature but not validated. The results of our study may help to distinguish between stable patients, in whom non-invasive rewarming can be attempted, and unstable patients. In the latter, extracorporeal rewarming should be considered, as the chances of survival may be high and prevail over the risk of complications.

Several definitions of hemodynamic instability in accidental hypothermia exist. Systolic blood pressure < 90 mmHg is most commonly stated and reasonable prehospital estimate of cardiocirculatory instability, but for in-hospital decisions, the minimum sufficient circulation for a patient in severe hypothermia (e.g., < 28 °C) has not been defined [3]. Attempts have been made to use lower systolic blood pressure values in clinical practice, and SBP ≤ 60 mmHg on admission along with severe arrhythmias may also be considered as a surrogate of circulatory instability and a threshold for ECLS [9, 10]. In the 5-A model predicting the risk of in-hospital mortality, hemodynamic instability was defined as an SBP of ≤ 60 mmHg, unmeasurable values, and cardiac arrest [11]. In the recent ICE-CRASH trial, severe AH was defined as unmeasurable blood pressure or systolic blood pressure of 60 mmHg or less [12]. In the Hypothermia Outcome Score SBP ≤ 70 mmHg is defined as “low systolic blood pressure” and is associated with an increased risk of death [13]. Two other studies identified MAP cut-offs of 80–90 mmHg to define high-risk populations for hypothermic death [14, 15]. However, it is not specified whether these lower limits were chosen by the authors arbitrarily or were based on clinical evidence.

Besides the debate on blood pressure values, a group of patients in whom blood pressure values could not be measured can be identified. Unmeasurable systolic blood pressure should be interpreted as an alert signal indicating the severity of hypoperfusion. In such a case, one recognizes “profound hypotension”, and PEA should be excluded [16]. However, commonly used devices for non-invasive blood pressure measurement called DINAMAP (device for indirect non-invasive measurement of mean arterial pressure), have been developed on the basis of the oscillometric method where systolic and/or diastolic blood pressure values may be obtained indirectly through manufacturer-owned extrapolation algorithms, whereas mean arterial pressure is a “direct” and most precise measurement [17]. Failure to display systolic and/or diastolic blood pressure value may be caused due to flaws in the extrapolation algorithms and artifacts. It should be noted that in our study, only six patients had reported non-measurable blood pressure, half of whom survived—this prompts reflection on the triaging the patient as a “not able to survive”.

The dichotomous approach in severely hypothermic non-CA patients in whom one group should be treated with ECLS and another with conventional rewarming methods, based only on the SBP threshold, is troublesome. Even SBP > 90 mmHg is not always sufficient to ensure adequate blood flow and organ perfusion. Our previous studies highlighted the important role of the acid–base balance parameters and the lactate concentration in the prognosis of victims of accidental hypothermia rewarmed with ECLS [18, 19]. Fluctuations of these parameters are secondary to organ perfusion pressure and require further investigation. In our study, paCO2 differed significantly between the deceased and survivor groups, but in both univariate analysis and multivariate modeling, it was not a parameter that was retained. On the other hand, BE was identified as a variable not significantly related to the outcome but making a significant contribution in the presence of other variables. Understanding these subtle changes requires further research into the pathophysiology of metabolic changes in hypothermia.

Factors that can significantly affect a patient's prognosis are their comorbidities and frailty. Since co-morbidities are usually unknown at initial management of many hypothermic patients (homeless people, unconscious, no personal identification), this parameter is useless as a risk factor at hospital admission. Therefore, in our analysis this element was excluded as a clinically relevant risk factor. By contrast, identifying where the patient was found may be crucial in assessing prognosis and implementing treatment strategies. This might result from several reasons. People found indoors are often older and more socially isolated. Ageing and lack of regular medical care may lead to delayed diagnosis and treatment of chronic diseases, consequently leading to their progression. Therefore, the exposition for indoor hypothermia may be prolonged but the cooling rate is slower than in the open air. These factors combined lead to a depletion of the body's (initially limited) compensatory capacity increasing the risk of complications and eventually death.

The present study has several strengths. This is the first study to provide a rationale for the use of 90 mmHg SBP threshold as a hemodynamic instability criterion. Second, the model is based on a homogeneous group of severely hypothermic patients with preserved circulation. Third, a simple prediction model based on only three parameters with high discrimination value after adjustment of the cutoff point of each parameter is provided.

## Limitations

The main limitation of our study is its retrospective and multicenter design with no uniform protocol for the management of a hypothermic patient. The applied method of data collection could induce selection bias. Due to the nature of hypothermia, studies involving this population tend to be underpowered. Also, in our study the size of the population was relatively small. Therefore, results of multivariable analyses should be interpreted with caution. Blood pressure measurements were made using various methods and devices. Due to incomplete data, the duration of cardiac arrest was not analyzed, and only the fact of rescue collapse was included in the regression modeling. For similar reasons, the catecholamine doses, mechanical ventilation parameters, iv fluid volume (and temperature) were not analyzed. Some of the data including comorbidity before hospitalization may be subject to error. We kept patients with preserved circulation but unmeasurable blood pressure in the analysis, as this is a possible clinical scenario.

## Data Availability

The data that support the findings of this study are not openly available due to reasons of sensitivity and are available from the corresponding author upon reasonable request. Data are located in controlled access data storage at International Hypothermia Registry (https://hypothermia-registry.org) and Hypothermia Life Support in Poland Registry (https://rejestrhipotermii.ujk.edu.pl).
